# Real-World Airborne Sound Analysis for Health Monitoring of Bearings in Railway Vehicles †

**DOI:** 10.3390/s26061947

**Published:** 2026-03-20

**Authors:** Matthias Kreuzer, David Schmidt, Simon Wokusch, Walter Kellermann

**Affiliations:** 1 Multimedia Communications and Signal Processing, Friedrich-Alexander-Universität Erlangen–Nürnberg (FAU), 91058 Erlangen, Germany; walter.kellermann@fau.de; 2 Siemens Mobility GmbH, 90441 Nürnberg, Germany; schmidtdavid@siemens.com (D.S.); simon.wokusch@siemens.com (S.W.)

**Keywords:** bearing fault, airborne sound analysis, MFCCs, train vehicles

## Abstract

In this paper, the task of detecting bearing faults in railway vehicles during regular operation by analyzing acoustic (airborne sound) data is addressed. To that end, various features are studied, among which the Mel Frequency Cepstral Coefficients (MFCCs) are best suited for detecting bearing faults by analyzing airborne sound. The MFCCs are used to train a Multi-Layer Perceptron (MLP) classifier. The proposed method is evaluated with real-world data for a state-of-the-art commuter railway vehicle in a dedicated measurement campaign. Classification results demonstrate that the chosen MFCC features allow for reliable detection of bearing damages, even for damages that were not included in training.

## 1. Introduction

Bearings are vital components in rotating machinery, e.g., the induction motors of trains. Bearing damages can have severe consequences as they may cause a complete failure of the machine and thus cause undesired costs and downtime. Hence, the early and reliable detection of bearing damages is of great importance. Since the manual inspection of bearings is often an intricate and cumbersome task due to their difficult accessibility, non-invasive condition monitoring techniques are preferred.

In principle, structure-borne sound (vibration) and airborne sound are suitable for non-invasive condition monitoring of bearings. Over the past decades, mainly structure-borne sound that can be captured by acceleration sensors on the housing of the machine under inspection has been investigated. This is due to the fact that localized faults at one of the four main bearing components, i.e., the inner race, the outer race, the cage, and the rolling elements, lead to periodic excitations that alter the typical vibration signature of the machine. For detecting these alterations, structure-borne sound has been analyzed by employing envelope analysis, signal decomposition, and filtering techniques [1,2]. More recently, there has been a surge in the popularity of end-to-end Deep Neural Network (DNN)-based methods for vibration fault detection [3,4,5], particularly in the realm of bearing fault detection.

Although the analysis of structure-borne sound has been well-studied and has proven to be very effective for the detection and classification of bearing damages, this approach has certain drawbacks. For one, the acceleration sensors need to be mounted in close proximity to the bearing that is to be monitored. While this can be rather easily ensured in laboratory setups, it proves to be much more difficult in practical scenarios, e.g., the monitoring of bearings in the induction motors of railway vehicles. In such a scenario, the sensors need to be mounted on the bogie of the train, where space is limited, and safety regulations have to be met. This makes the retrofitting of sensor equipment for already existing vehicle platforms especially challenging. For such applications, less intrusive methods are preferable.

Methods for detection and classification of bearing faults using airborne sound have the potential to overcome some of the drawbacks of structure-borne sound-based monitoring. Microphones are less intrusive than acceleration sensors since they do not have to be placed directly on the component that is to be monitored but only in its vicinity. Therefore, the specifications regarding the placement of sensors are less strict, and microphones can also potentially be used to monitor multiple components simultaneously. Further, the sensing cost for microphones is usually significantly lower [6]. Finally, a multi-modal classification, i.e., the combination of airborne sound data and structure-borne sound data, can lead to more reliable classification results.

The classification of bearing faults by analyzing airborne sound data has already been addressed in [7], where a bearing fault feature extraction approach is proposed that combines Adaptive Variational Mode Decomposition (AVMD), an Improved Multiverse Optimization (IMVO) algorithm, and Maximum Correlated Kurtosis Deconvolution (MCKD) to subsequently identify fault features in the envelope spectrum of airborne sound. For a related application, the Variational Mode Decomposition (VMD) is used for denoising in [8]. In [8], which aims at the detection of cylinder misfires or blocked air inlets in a diesel engine, MFCCs are extracted from the denoised signals, which are then used to train a long short-term memory (LSTM) network, which acts as the classifier. Another signal decomposition technique, the Fourier decomposition method (FMD), is applied in [9] for the task of classifying bearing faults. The kurtosis of both the time-domain signal and its envelope are then extracted from the decomposed signals and used as features for training a random forest classification algorithm. Whereas the approaches in [7,8] rely on extracting hand-crafted features, the task of identifying discriminant features was handed to a Stacked Auto-Encoder (SAE), which operates on the raw spectrograms of sound signals. Moreover, in [10,11,12,13,14], features are extracted from the frequency-domain representations of sound signals, which are then fed into machine learning (ML)-based classifiers, e.g., k-Nearest Neighbors (k-NN), Support Vector Machine (SVM), and MLP, to classify bearing faults. The task of classifying bearing faults by analyzing sound signals and applying DNN-based methods is addressed in [9,15,16].

To the authors’ knowledge, all the above methods have only been evaluated in controlled laboratory settings using test benches in the absence of strong interferers. Furthermore, the proposed classifiers were not assessed with unfamiliar fault conditions, which are likely to occur in real-world situations. In contrast, this study, which expands upon our work in [17], analyzes the potential of analyzing airborne sound for the detection and classification of bearing faults in induction motors and gearboxes in a very challenging measurement environment: a modern commuter railway vehicle during regular operation. Although end-to-end DNN-based classification approaches have proven to be very effective for a variety of classification tasks, they exhibit certain drawbacks. Especially in practical scenarios in which you want to perform on-board condition monitoring, DNN-based approaches are not feasible due to memory and processing limitations. Further, in order to properly train DNNs, large amounts of training data are usually required. Otherwise, DNNs are prone to overfit. Consequently, feature-based approaches are still sought after as they require less processing power, consume less memory and allow for an easier interpretation.

To that end, various features from the time domain and the frequency domain as well as features for acoustic scene classification tasks are evaluated in an experimental study. Further, various classifiers will be evaluated using the best-performing feature set to determine the optimal bearing fault detection approach for the evaluated scenario. In the end, it will be demonstrated that MFCCs are highly effective features, and when they are combined with a relatively simple MLP, they enable the detection of bearing faults in a practical condition monitoring setup, even when encountering unseen fault conditions.

The remainder of this paper is structured as follows. In Section 2, the considered scenario is described diligently. Specifically, the railway vehicle is described in Section 2.1, the placement of the sensors is addressed in Section 2.2, the investigated bearing damages are described in Section 2.3, and the data acquisition process is presented in Section 2.4. Thereafter, the proposed bearing fault classification approach is discussed. First, the suitability of airborne sound for the classification of bearing faults is shown in Section 3.1. After this, the feature selection process and the choice of classifier are discussed in Section 3.2 and Section 3.3, respectively. The presented bearing fault classification approach is then evaluated on seen and unseen damages in Section 3.4. Lastly, conclusions are drawn in Section 4.

## 2. Scenario: Experimental Setup and Field Measurements

First, descriptions of the railway vehicle, the placement of the sensors, the bearing damages and the data acquisition process are given as background for our subsequent analysis.

### 2.1. Description of the Railway Vehicle

**Figure 1:**
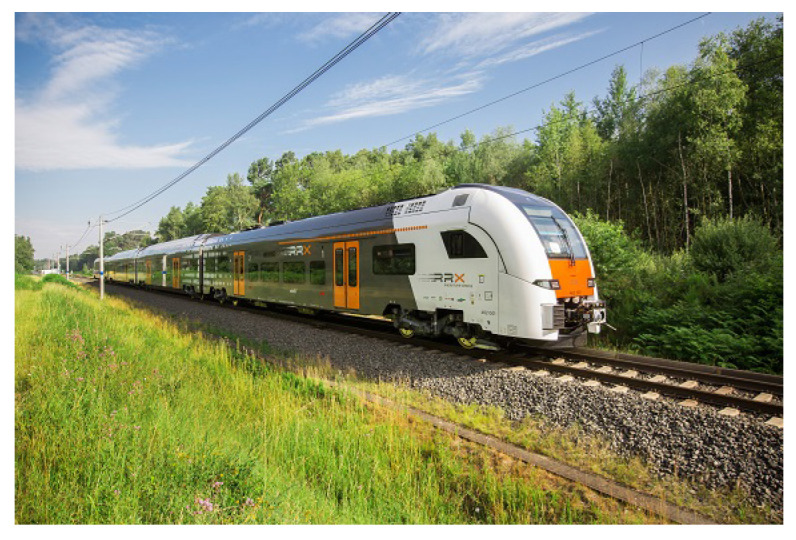
Photograph of the commuter rail vehicle of the type Desiro HC RRX that was equipped with measurement equipment to obtain the real-world data. Reprinted with permission from [17].

**Figure 2:**
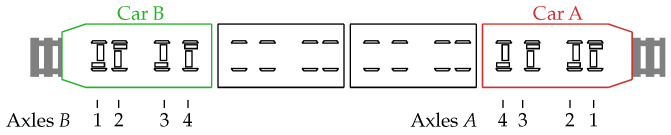
Schematic view of the railway vehicle. Two cars, i.e., Car A and Car B, were equipped with condition monitoring equipment. The damaged bearings were installed in Car A, whereas Car B was only equipped with healthy bearings and hence serves as a reference for the healthy state. Reprinted with permission from [17].

For our investigations, a state-of-the-art commuter railway vehicle of the type Desiro HC RRX (Rhein-Ruhr-Express) [18] as depicted in Figure 1 is considered. For acquiring a sufficient amount of realistic bearing fault data, the railway vehicle was equipped with damaged bearings and a multitude of sensors. For the measurement campaign, two cars were monitored on two separate test trips between two cities on regular railway tracks with a duration of approximately 4 h each, conducted on public railway infrastructure under real operating conditions. Two of the four train cars, i.e., Car A and Car B (cf. Figure 2), were equipped with sensors, i.e., acceleration sensors, temperature sensors, microphones, etc., but in the following only the microphone data is considered. A drivetrain consisting of a motor and a gearbox is positioned at each of the four axles (cf. Figure 4). The axles are referred to as Bi and Ai with i∈{1,2,3,4} for Car B and Car A, respectively. The measurements of Car B serve as reference for the healthy state of a bearing since this car exhibits only healthy bearings, whereas Car A was equipped with damaged bearings. The damaged bearings will be described in more detail in Section 2.3.

### 2.2. Placement of the Microphones on the Railway Vehicle

**Figure 3:**
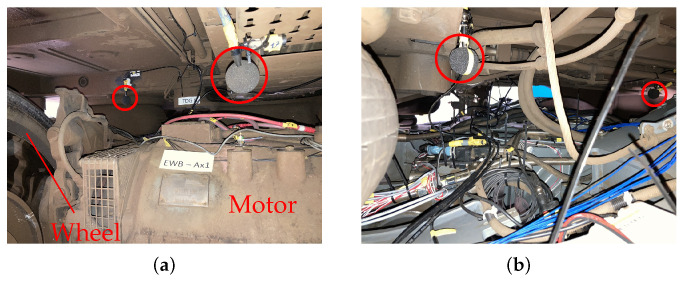
Placement of the microphones (○) on the rail vehicle. A single microphone is placed above each drivetrain component by attaching it to the bottom of the railway car. Subfigure (**a**) depicts the microphone above the motor at the first axle of Car B (foreground) and Subfigure (**b**) shows the microphone above the motor from a different perspective. Subfigure (**b**) also shows the measurement equipment for acceleration and current. Reprinted with permission from [17].

**Figure 4:**
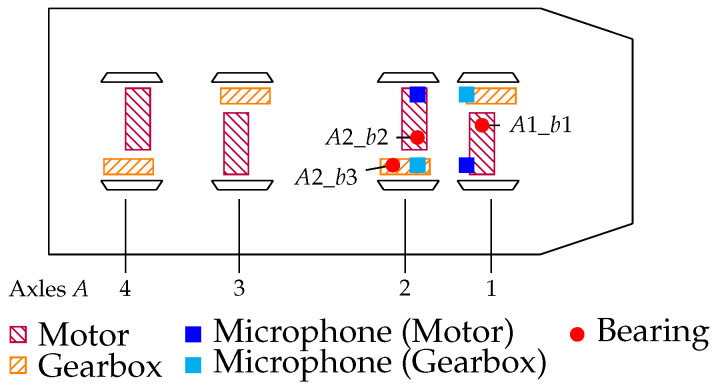
Schematic view of Car A. The first two drivetrains are equipped with acoustic sensors. A microphone is placed at a central position above each drivetrain component at Axle A1 and Axle A2. The microphones above the motors are depicted as ■ and the microphones above the gearboxes are depicted as ■. The positions of the damaged bearings are indicated by red circles (•). The microphone setup in Car B is identical. Reprinted with permission from [17].

As depicted in Figure 3, microphones were installed above every drivetrain component by attaching them to the bottom of the railway car. Consequently, the distance between the monitored component and the microphone is approximately 30cm. This was done for the first two axles of Car A and Car B. The locations of the microphones can be seen in Figure 4. In Figure 4, the microphone locations are marked by ■ and ■. The microphones marked by ■ are considered for classification tasks at the motor and the microphones marked by ■ are considered for classification tasks at the gearbox. Note that the microphones do not only capture the sounds that are caused by the bearings but also noise that is emitted from other components in the train bogie, e.g., brakes, dampeners, etc., or noise caused by the railway tracks.

### 2.3. Description of the Bearing Damages

As pointed out in Section 2, all of the investigated damaged bearings are installed in Car A. These bearing damages are summarized in Table 1 and are denoted by A1_b1, A2_b2 and A2_b3. The notation is explained as follows: the first identifier A1 in A1_b1 refers to the axle, i.e., Axle 1 in Car A, and the second identifier b1 refers to bearing b1 (cf. Figure 4 and Table 1). Hence, the corresponding healthy bearings in Car B are denoted as B1_b1, B2_b2 and B2_b3, respectively. Bearing A1_b1 represents a bearing fault in a very early stage, whereas A2_b2 represents a bearing with a fault in a slightly more developed stage. Bearing A2_b3 is in the gearbox (G) and represents a fault at a developed stage. The locations of the bearings and the fault types can be inferred from Table 1. Note that the considered bearing damages did not develop naturally but were introduced artificially.

### 2.4. Data Acquisition

Omni-directional electret microphones of the type ‘M 370’ [19] were employed to capture signals at a sampling frequency of 25.6kHz. The signals were segmented into non-overlapping frames, each containing 2048 samples (80ms). For the evaluation, only frames with a mean rotational frequency of the axle within the range of $42\;\mathrm{Hz}\leq {\bar{f}}_{r}\leq 45\;\mathrm{Hz}$ are considered, as this frequency range was most commonly observed during the measurements. Notably, there were no restrictions imposed regarding the applied torque, the mode of the power converter, or the driving direction. Consequently, approximately 28,000 frames were obtained for each microphone.

## 3. Bearing Fault Classification Approach

Now that the considered scenario has been introduced, the bearing fault detection approach based on the analysis of airborne sound is presented next. At first, the use of airborne sound data for fault diagnosis is motivated by highlighting the similarities between spectrograms obtained for airborne sound data and spectrograms obtained for structure-borne sound data in Section 3.1. In this paper, a two-stage procedure is followed to find the optimal approach for the classification of bearing faults by analyzing airborne sound data. In the first stage, the most suitable features for bearing fault classification using airborne sound are identified in Section 3.2 by benchmarking the different features using the same classifier. In the second stage, the performance is optimized further by finding the best-performing classifier for the previously selected feature set in Section 3.3. Finally, the proposed bearing fault classification approach is evaluated for different scenarios in Section 3.4.

### 3.1. Airborne Sound vs. Structure-Borne Sound

**Figure 5:**
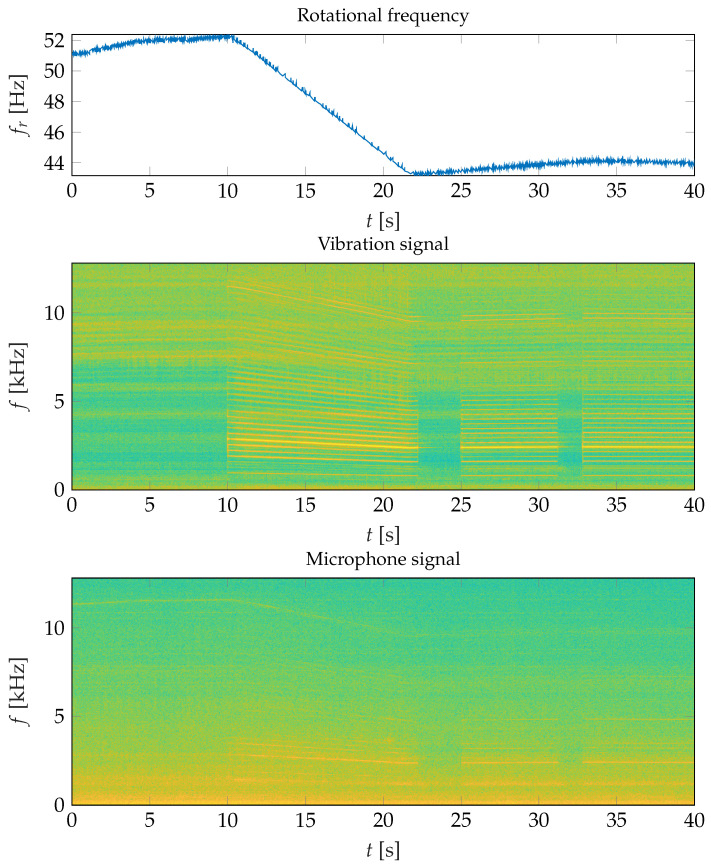
Rotational frequency (**top**), spectrogram of the vibration signal (**center**) and spectrogram of the microphone signal (**bottom**) of a healthy bearing. Reprinted with permission from [17].

To motivate the use of airborne sound data for bearing fault detection, we take a look at the following spectrograms: Figure 5 shows a rotational frequency curve (top figure) and the corresponding spectrograms for the structure-borne sound measured directly on the housing of the motor of Axle B1 in Car B (center figure) and the spectrogram for the airborne sound (bottom figure) that was captured by the microphone located at the motor at the same axle and on the same car, i.e., B1_b1. The rotational frequency curve exhibits a period of almost constant rotational speed, which is followed by a second period of constant rotational speed after a deceleration phase of approximately 10s. It can be observed that the spectrogram of the vibration signal is dominated by harmonics of the rotational frequency. These harmonics can be clearly observed in the spectrum over the entire frequency range for time periods when torque is applied. The spectrogram of the microphone signal reveals a similar structure, although it is less prominent compared to the vibration signal. While the vibration signal exhibits sharp horizontal lines representing harmonics of the rotational frequency across the entire frequency spectrum, only a few faint harmonics are discernible in the microphone signal, predominantly below 5kHz. Thus, it can be concluded that spectrograms generated for airborne sound signals share a resemblance with those for structure-borne sound signals, albeit with incomplete and less pronounced representation of components associated with periodic events in the motor.

**Figure 6:**
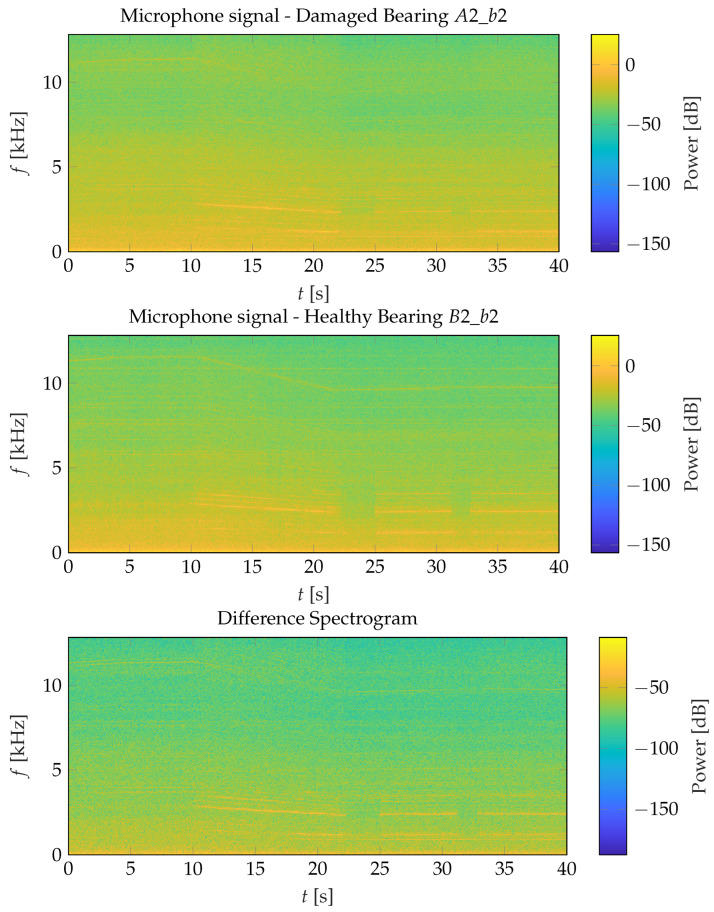
Spectrogram for the bearing damage at Axle 2, i.e., A2_b2, (**top**), spectrogram for the healthy bearing at Axle 2, i.e., B2_b2 (**center**), and difference spectrogram between spectrograms of the healthy and damaged bearing normalized to the average signal power of the reference (healthy) signal (**bottom**).

Figure 6 shows the power spectrograms of bearing damage A2_b2 (top) and the corresponding healthy reference microphone at Axle 1 in Car B, B2_b2 for the same time interval as in Figure 5. The spectrogram of A2_b2 appears as a noisier version of the healthy reference. In both cases, the dominant spectral component is located at $54\cdot f_{r}$. For instance, between 25s and 30s it occurs at approximately 54·44Hz=2376Hz, which coincides with the stator slot number of the motor. Compared to the healthy signal, the damaged spectrogram exhibits increased noise, particularly in the 4kHz to 7kHz range. The bottom subfigure of Figure 6 presents the difference power spectrogram between the healthy and damaged signals. Prior to subtraction, both spectrogram magnitudes were normalized to the average power of the healthy reference. The difference spectrogram indicates that the bearing damage does not introduce new distinct spectral components. Instead, it alters the signal power at the dominant harmonics of the rotational frequency.

Consequently, harmonics from bearings are recognizable in the sound signal that is captured in its proximity, and thus, changes w.r.t the condition of bearings should be detectable using adequate features.

### 3.2. Feature Evaluation and Selection

Following the above, we now investigate which features are best suited for measuring these alterations and thus identifying bearing faults successfully. To this end, we consider various statistical features from the time and the frequency domains. The selection of features is based on two criteria: (i) their ability to capture amplitude variations, impulsiveness and spectral energy redistribution caused by bearing damage, and (ii) their established use in audio signal analysis and bearing fault diagnostics reported in the literature. This grouping serves as a structured and interpretable comparison of conceptually different feature types. However, it does not restrict the final feature selection, as in a subsequent step all features are jointly evaluated using established feature-ranking techniques to determine the most informative subset independent of the initial grouping.

From the time domain we consider a set of well-known statistical features that are summarized in Table 2, where x[k] denotes the time-domain microphone signal at time instant *k*, which is obtained after sampling the continuous-time microphone signal with sampling frequency $f_{s}=25.6\;\mathrm{kHz}$. This sampling frequency was not chosen for a specific reason, and the decision was made by our industry partner. The features listed in Table 2 form the first feature set, which is referred to as *TD* in the following. These features were applied for the classification of bearing fault damages, for example, in [10,20,21,22].

In addition to the time-domain features listed above, we also consider various features from the frequency domain, e.g., spectral centroid and spectral kurtosis, which are computed from the frequency-domain representation of x[k], i.e., X[μ]. For the discrete-time signal x[k] with *K* samples of length *M*, Discrete Fourier Transform (DFT) can be defined as follows:

(1) $$\begin{array}{c} X\left[\mu \right]={\mathrm{DFT}}_{M}\left\{x\left[k\right]\right\}=\sum_{k=0}^{K-1}x\left[k\right]e^{-j\frac{2\pi }{M}k\mu }, \end{array}$$

where μ and *M* denote the frequency bin index and the length of the DFT, respectively [23]. The considered features derived from the frequency-domain representation X[μ] are listed in Table 3. These features form the second feature set, which is referred to as *FD*. In the literature, these features were applied for the task of classifying bearing faults, for example, in [10,24,25].

Localized bearing faults lead to periodic excitations which are caused by rolling elements every time they roll over a defect. Consequently, localized faults can be linked to characteristic frequencies [1] that are solely determined by the rotational frequency and the geometry of the bearing. Therefore, the amplitudes at multiples of the characteristic fault frequencies in the envelope magnitude spectrum of the microphone signals are also considered as features. These features are computed as amplitudes at multiples of the characteristic fault frequencies as follows:

(2) $$\begin{array}{c} AMP_{fault}=\sum_{i=1}^{5}\left|X_{env}\left[i\cdot \mu_{fault}\right]\right|\\ \mathrm{with}\;\mu_{fault}\in \left\{\mu_{\mathrm{BPFO}},\mu_{\mathrm{BPFI}},\mu_{\mathrm{CA}},\mu_{\mathrm{RE}}\right\}, \end{array}$$

where $X_{env}\left[\mu \right]$ denotes the envelope spectrum (cf. [1]), which is obtained by applying the Hilbert transform [23], and $\mu_{fault}$ is the frequency bin index corresponding to the characteristic fault frequencies $f_{\mathrm{BPFO}}$, $f_{\mathrm{BPFI}}$, $f_{\mathrm{CA}}$ and $f_{\mathrm{RE}}$ for a localized fault in the outer race, the inner race, the rolling cage and the rolling elements, respectively. To determine the characteristic fault frequencies, we refer to [1]. Typically, these fault-frequency-based features are used in the context of structure-borne sound analysis. According to Equation (2), a four-dimensional feature vector is formed, which is referred to as *ENV* in the following. In [26] it was shown that bearing faults could be reliably detected with state-of-the-art features for acoustic scene classification tasks that were extracted from structure-borne sound data. More specifically, the first 13 MFCCs were computed for vibration signals and were used as features to train a One-Class SVM where accuracies above 96% could be obtained for laboratory data. MFCCs are state-of-the-art features for acoustic scene classification and speaker recognition tasks [27,28,29,30,31,32], as they allow for a compact representation of the spectrum of a signal by combining the cepstrum with a scaling of the frequency on the Mel scale [33]. The MFCCs $c_{\mu }$ for time frame *n* are computed as

(3) $$\begin{array}{c} c_{\mu }=\sum_{i=1}^{K}\log X_{i}\left[n\right]\cos\left(\frac{\pi (2i-1)\mu }{2K}\right),\;\mu =1,\ldots ,K, \end{array}$$

where $X_{i}\left[n\right]$ denotes the spectral energies of time frame *n* with i=0,…,K−1, which are computed as

(4) $$\begin{array}{c} X_{i}\left[n\right]=\sum_{\nu =0}^{N-1}g_{i\nu }{\left|\sum_{k=0}^{N-1}x\left[n-k\right]w\left[k\right]e^{-\frac{2\pi k\nu }{N}}\right|}^{2}, \end{array}$$

where x[k] are the time-domain input samples, w[k] is a window function and $g_{i\nu }$ are the samples of a triangular window sequence for weighting the ν-th frequency bin for the *i*-th channel of the Mel filterbank output, denoted by $X_{i}\left[n\right]$. For a more detailed description of the computational steps that are required for computing the Mel Frequency Cepstral Coefficients (MFCCs), refer to [33]. Further, for the remainder of this paper, the first 13 MFCCs with K=2048 are used as features if not explicitly stated otherwise.

**Figure 7:**
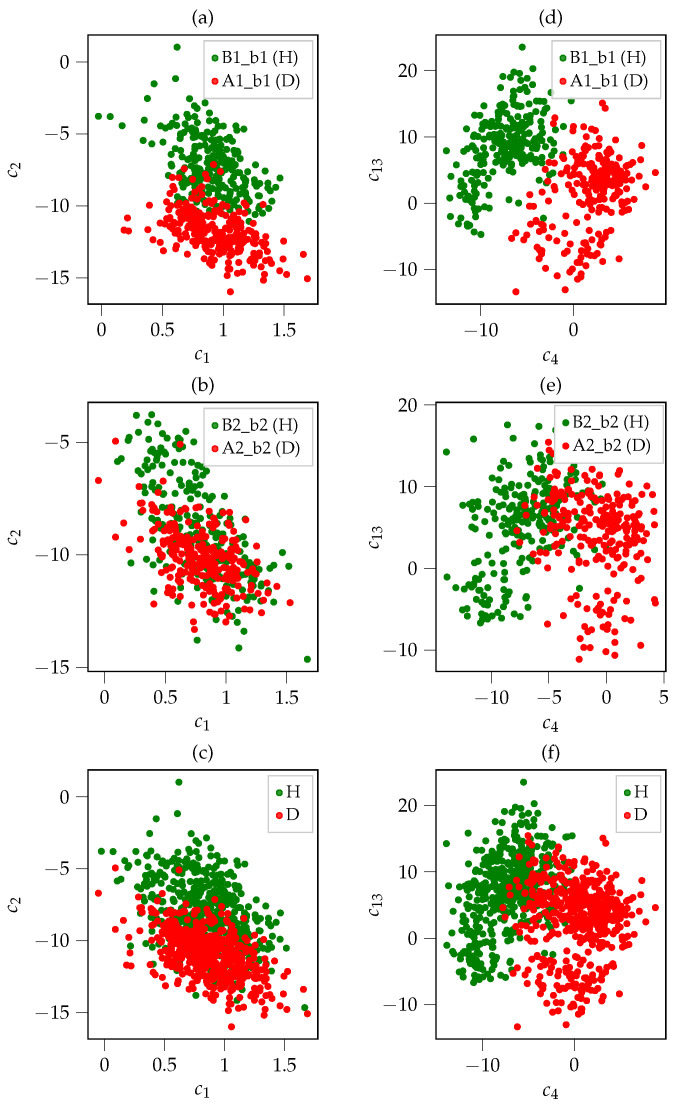
MFCC scatter plots for the microphone data of the motor. (**a**–**c**) show scatter plots for the values the 1st MFCC, i.e., $c_{1}$, on the x-axis and the values for the 2nd MFCC, i.e., $c_{2}$, on the y-axis, whereas (**d**–**f**) show scatter plots for the 4th MFCC, i.e., $c_{4}$, and the 13th MFCC, i.e., $c_{13}$. For (**c**,**f**), the data points for B1_b1 and B2_b2 and A2_b2 and A1_b1 were combined to H and D, respectively. Note that red data points originate from measurements of damaged bearings, whereas green data points originate from measurements of healthy bearings. Reprinted with permission from [17].

An indication of their appropriateness is provided by the following example: Figure 7 shows two-dimensional scatter plots for two exemplary combinations of MFCCs. Figure 7a–c depicts scatter plots in which the values of the first MFCC, i.e., $c_{1}$, are shown along the x-axis and the values for the second MFCC, i.e., $c_{2}$, are shown on the y-axis. Figure 7a shows the data points for the healthy bearing B1_b1 and the damaged bearing A1_b1, respectively, whereas Figure 7b shows the data points for B2_b2 and A2_b2. B1_b1 and B2_b2 denote the data that was obtained from the microphones above the motors at Axle B1 and Axle B2 of Car B, respectively (cf. Figure 2). For Figure 7c, the data points for B1_b1 and B2_b2 and A2_b2 and A1_b1 are combined for the labels H (healthy) and D (damaged), respectively. For Figure 7d–f, a different combination of MFCCs is used: the values for the fourth MFCC, i.e., $c_{4}$, are given along the x-axis and the values of the thirteenth MFCC, i.e., $c_{13}$, are plotted on the y-axis. Again, the data points for B1_b1 and B2_b2 and A1_b1 and A2_b2 are combined for the labels H and D, respectively, in Figure 7f.

**Figure 8:**
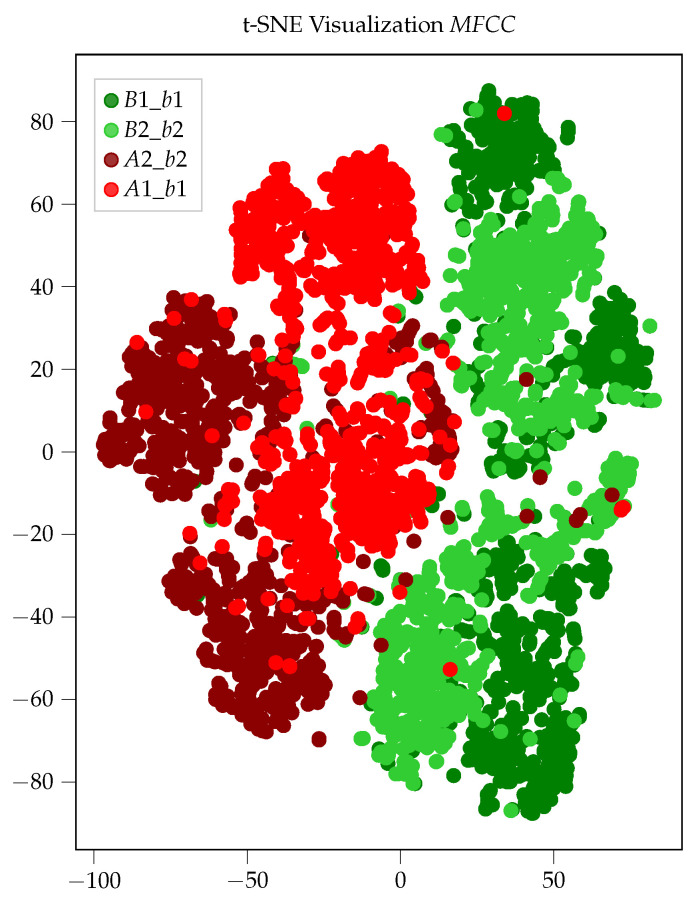
t-SNE visualization for *MFCC* feature set.

**Figure 9:**
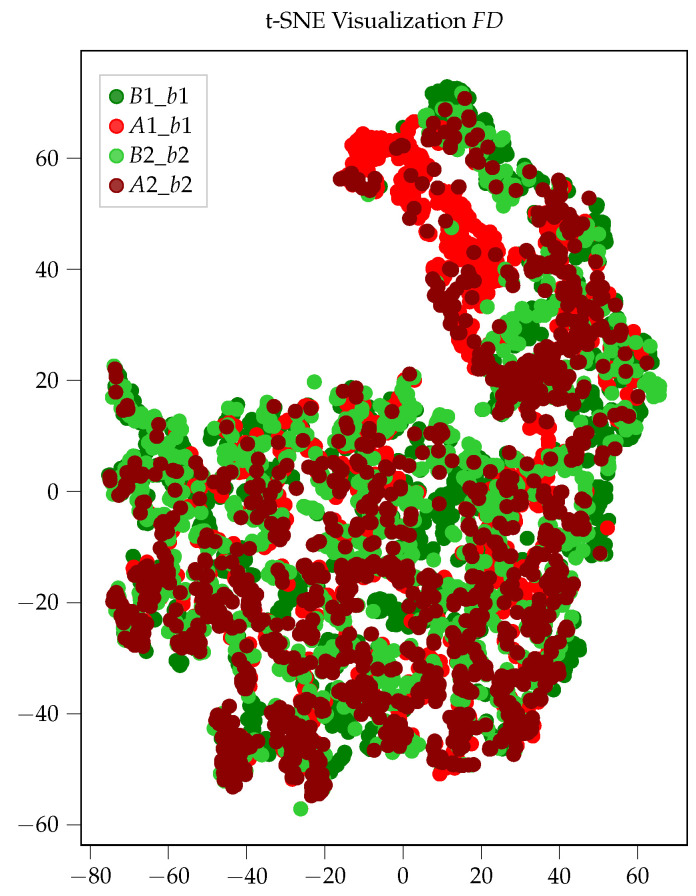
t-SNE visualization for *FD* feature set.

Figure 7a–f shows that the point clouds for the two classes, healthy (H) and damaged (D), only overlap to a small extent in these two exemplary two-dimensional subspaces. Hence, it is already possible to roughly discriminate between healthy and damaged samples in these two-dimensional subspaces. This is a clear indication that the MFCCs are well-suited for the classification of bearing faults using airborne sound. Since we cannot visualize the 13-dimensional subspace, we use the t-Stochastic Neighborhood Embedding (t-SNE) [34] technique for visualization purposes. t-SNE is a dimensionality reduction technique that allows the visualization of high-dimensional data in a lower-dimensional space, e.g., two-dimensional. The resulting scatter plot for the two-dimensional t-SNE visualization is shown in Figure 8. Here, the features for the healthy bearings B1_b1 and B2_b2 are depicted in dark and light green, whereas the features for the damaged bearings A1_b1 and A2_b2 are depicted in dark and light red. From Figure 8, we can see that the green and red scatter points form distinct clusters with only a few outliers. This further emphasizes the appropriateness of MFCCs as features for the detection of bearing faults by analyzing airborne sound. The situation is quite different for the other considered feature sets, i.e., *TD*, *FD* and *ENV*, as no distinct point clouds can be observed for healthy and damaged bearings as is the case in Figure 8. The t-SNE visualization for the *FD* feature set that is shown in Figure 9 serves as a representative example. After observing Figure 9, it becomes apparent that the data points for the healthy bearings B1_b1 and B2_b2 and the damaged bearings A1_b1 and A2_b2 do not lie separated from each other in the two-dimensional subspace but overlap and form one big point cloud. Similar pictures were obtained for the other two feature sets, i.e., *TD* and *ENV*. This indicates that in comparison to MFCCs, the statistical features from the time domain (*TD*) and the frequency domain (*FD*) as well as the bearing-fault-frequency-based features (*ENV*) are not equally well suited for the detection of bearing faults in the motors of railway vehicles using airborne sound.

Figure 7, Figure 8 and Figure 9 already hinted at the superiority of MFCCs as features, but this is further evidenced by the following classification results. The introduced feature sets are used to train an SVM [35] and are evaluated in a challenging scenario with unseen data. In particular, the data from the first axles of Car A and Car B are used for training, whereas the data from the second axles of Car A and Car B are used for testing. Consequently, B1_b1 serves as reference for the healthy class (H) and A1_b1 serves as reference for the damaged class (D) in the training set, and B2_b2 and A2_b2 (cf. Figure 3 and Figure 4) represent the healthy and damaged class in the test set, respectively. This scenario is challenging, as the classifier is confronted with data from bearings that were not included in the training. Further, the fact that the bearing fault in the test, i.e., A2_b2, is an outer race fault whereas the bearing damage in the training set, i.e., A1_b1, is an inner race fault further adds to the difficulty of the classification task.

**Figure 10:**
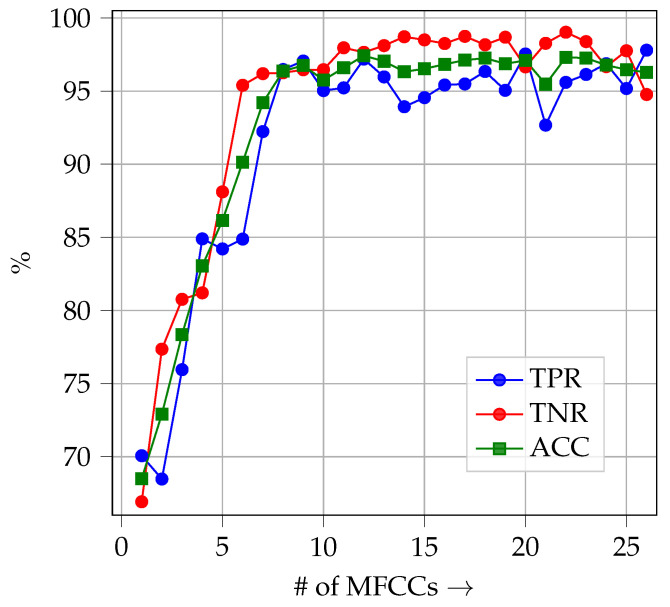
TPR, TNR and ACC depending on the number of considered MFCCs for a binary classification problem with unseen bearing damage A2_b2 in the test set.

As the classifier, an SVM with Radial Basis Function (RBF) kernel [35] is utilized whose parameters are optimized following a 5-fold cross-validation approach on the training data. The training and test sets each consist of 50,000 frames of length 2048. The results are summarized in Table 4. In Table 4, the True Positive Rate (TPR) indicates how well the healthy class is predicted, whereas the True Negative Rate (TNR) indicates how well the damaged class is predicted by the classifier. *ACC* denotes the overall accuracy. A good classifier exhibits both a high TPR and a high TNR as this shows that the classifier is able to predict both classes equally well and is not biased towards a single class. From Table 4 it can be inferred that the three feature sets *TD*, *FD* and *ENV* do not allow for a reliable classification of unseen damages at all, as the overall accuracies are 60.71%, 54.78% and 62.19%, respectively. The obtained accuracy values demonstrate that these features cannot reliably classify bearing faults if they are extracted from raw microphone signals. The combination of *TD*, *FD* and *ENV* to a single feature vector of length 21 yields an improved overall accuracy of 80.16%. Thus, it has been demonstrated that these feature sets are not well-suited for the classification of unseen bearing damages. However, with an overall accuracy of 94.05, the *MFCC* feature set is by far the best-performing feature set. The bearing damage can be accurately predicted as the TNR is 94.52%. With a TPR of 93.57%, the accuracy for the healthy class is slightly worse, yet the healthy class can still be very well predicted. The classification performance depending on the number of MFCCs that are considered for classification for the same scenario is illustrated in Figure 10. From Figure 10 it can be gathered that already accuracies above 95% can be gathered with the first six MFCCs [36]. Yet, the classification performance does not improve by considering more than 13 MFCCs for decision making, which coincides with empirical findings for speaker recognition tasks [36]. Thus, choosing the first 13 MFCCs as a feature set is a reasonable choice.

Evidently, the performance of the *MFCC* feature set is far superior to all other feature sets. Due to this large difference, there is no reason to assume that any of the other feature sets would outperform the *MFCC* if another classifier was chosen.

To corroborate our feature selection even further, we also consider the following feature-ranking methods:

- **Relief:** A feature ranking method that estimates the importance of each feature based on how well it discriminates between instances that are similar and those that are different. This is achieved by computing the difference of feature values between the nearest instances of the same class (near-hit) and the nearest instances of the other class (near-miss). Features are deemed to be more important if the difference for the near-miss is larger than for the near-hit [37].
- **Minimal-Redundancy-maximal-Relevance (mRMR):** Features are ranked by evaluating their relevance to the target variable, i.e., the class label, and their redundancy with other features. Relevance and redundancy are measured based on mutual information. Features are then selected to maximize relevance while minimizing redundancy. The result is a ranked list of features [38].
- **Decision trees (DTs):** The importance of features can also be estimated with decision trees. How much each feature contributes to the overall predictive accuracy of the decision tree is evaluated. Features with higher importance values are deemed more influential in making predictions.
- **Sequential feature selection (SFS):** This method systematically evaluates different combinations of features by iteratively adding a feature to the feature set based on how the addition of this specific feature affects a defined criterion, e.g., the overall accuracy.

The feature ranking results that were obtained for the feature ranking techniques listed above are summarized in Table 5. Every column lists the 13 best features as they were identified by the respective feature ranking method. The last row in Table 5 lists the accuracy values that were obtained for the mixed feature sets for the same scenario that was already considered in Table 4. After studying Table 5, it becomes evident that all four feature-ranking techniques clearly identify the MFCCs, which are denoted as $c_{\mu }$, as the most important features for the reliable classification of unseen damages, since each feature set consists mainly of MFCCs. Remarkably, the mixed feature sets are not able to outperform the pure MFCC feature vector, as evidenced by the results in Table 5 and Table 4, although data from all bearings was considered for each feature-ranking technique. Thus, after considering all the investigations above, it can be clearly stated that the MFCCs are the best suited features for the task at hand. Note that with the data used, the feature ranking methods should ideally yield the set of 13 MFCCs as optimum features or produce a feature vector that outperforms the MFCC feature vector. The reason for the given results remains to be investigated.

### 3.3. Classification

**Figure 11:**
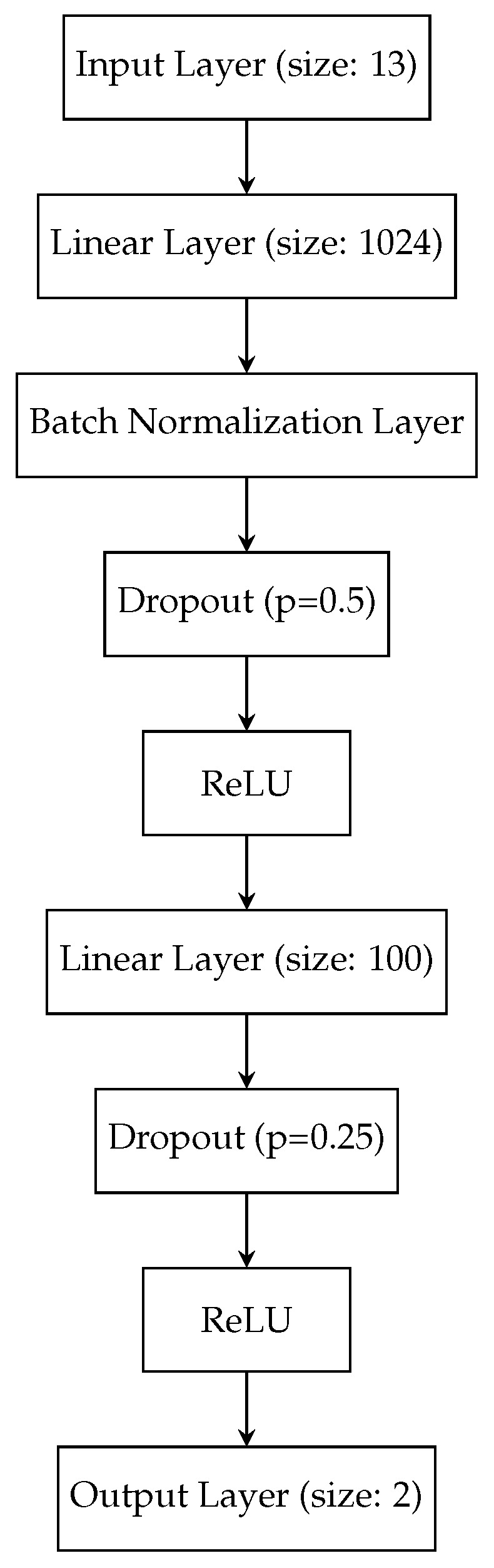
Architecture of the proposed MLP classifier.

Since it has been demonstrated that the MFCCs are the best-performing features with a commonly used classifier, it is now investigated in the next step whether the classification performance can be improved even further by pairing the MFCCs with another classifier. To this end, various supervised machine learning classifiers are evaluated for the scenario that was already evaluated in Section 3.2. For our comparative study, we consider the following classifiers: k-NN [39], SVMs [40] with both linear and polynomial kernels, Decision Tree [20], Random Forest [41], AdaBoost [42], Naive Bayes [43,44], Linear Discriminant Analysis (LDA) [45], Quadratic Discriminant Analysis (QDA) [46] and MLPs [21]. At this point, we forego the introduction of each classifier as it would exceed the scope of this paper, but refer to [35] for a more detailed description and to [47] for implementation details. For the k-NN classifier, we consider two different values, i.e., k=5 and k=10. Just like in Table 4, we include the SVM in our evaluation but now consider an SVM with a linear kernel and an SVM with a polynomial kernel with a degree of 3 instead of the RBF kernel. We also consider a simple MLP classifier, i.e., MLP (baseline), which consists of one hidden layer with 100 neurons, and our proposed MLP classifier whose architecture is shown in Figure 11. The proposed MLP architecture consists of two hidden layers with 1024 and 100 neurons, respectively. The first hidden layer is followed by a batch normalization layer, a dropout layer with dropout probability p=0.5 to decrease the risk of overfitting and a Rectified Linear Unit (ReLU) activation function. A second dropout layer with dropout probability with p=0.25 is added after the second hidden layer of size 100. Then, another ReLU is added before the output layer of size 2. The classification results for all considered classifiers are summarized in Table 6. Again, the TPR, the TNR and the overall accuracy (ACC) are considered in our evaluation. Considering Table 6, it can be stated that MFCCs are well-suited features independent of the classifier, as the overall accuracy for 9 of the 12 considered classifiers is above 91% and the lowest accuracy is still above 84% for the rather simple *Naive Bayes* classifier. With the k-NN classifier, which is a popular choice due to its simplicity, accuracies of 95.92% and 96.31% can be obtained for k=5 and k=10, respectively. Although the accuracy could be improved by increasing the value for *k* from 5 to 10, choosing k>10 did not improve the accuracy any further. After comparing the three different SVMs—*SVM (RBF Kernel)* (cf. Table 4), *SVM (lin. Kernel)* and *SVM (poly. Kernel)*—it can be stated that the highest accuracy is achieved by *SVM (lin. Kernel)* with an accuracy value of 94.46. However, when inspecting TPR and TNR, it can be noticed that the classifier is biased towards TNR, which is obvious from the 6 percentage point difference relative to TPR. While the overall accuracy for SVM with RBF kernel is 0.41 percentage points lower, the difference between TPR and TNR is not as significant, i.e., 93.57% vs. 94.52%, which is preferable. However, the polynomial kernel with a degree of 3 is the worst-performing kernel choice, as the overall accuracy is 86.62%. For the decision-tree-based classifiers—*Decision Tree*, *Random Forest* and *AdaBoost*—accuracies of 84.84%, 91.16% and 93.87% are achieved. As *Random Forest* and *AdaBoost* are ensemble learning methods that combine more than one decision tree for decision making, it is natural that better accuracies are obtained. Very good results can also be obtained by the discriminant analysis classifiers, *LDA* and *QDA*, as accuracy values of 95.15% and 96.77% can be observed, respectively. With the *MLP (baseline)* classifier, an accuracy of 95.02% is also achieved. Yet, with our proposed MLP classifier, i.e., *MLP (proposed)*, the accuracy can be increased by over 2 percentage points to 97.04%. Therefore it can be stated that by using the MFCCs as features, good classification results can be obtained with a variety of classifiers, which further supports their aptitude. In conclusion, it can be stated that leveraging MFCCs as features yields consistently superior classification results across various classifiers, which underscores their efficacy and suitability for the task. Since our proposed classifier yielded the best results for the considered scenario, our further investigations are based on *MLP (proposed)* and the MFCCs as features. Please note that end-to-end DNN-based approaches have not been completely excluded from our investigation, as Convolutional Neural Networks (CNNs) were also considered. In particular, ResNet [48] models of varying model complexity were trained to automatically extract features from the raw microphone data. However, the considered models did not outperform the feature-based approach.

### 3.4. Experiments

To complete our experimental study, we investigate some more classification scenarios with real-world data. In our evaluation, we distinguish between scenarios with seen bearing damages and unseen bearing damages. In Section 3.2 and Section 3.3, we already considered a challenging scenario with unseen damages. Although scenarios with unseen damages are highly relevant for practical applications, scenarios involving seen damages are predominant in the literature. In these scenarios a random split is performed on the available data to generate the training and test data. Thus, the classifier has “seen” all bearings in some capacity in training.

#### 3.4.1. Classification with Seen Damages

**Figure 12:**
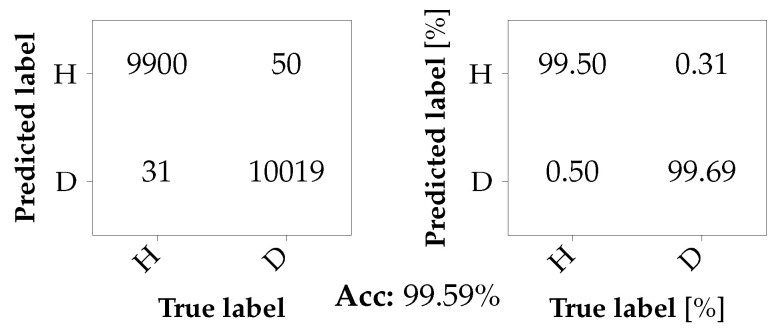
Confusion matrix for a binary classification problem at the motor with seen damages.

For the first classification task, a binary classification at the engine is considered. The classifier is trained with data from the first two axles of Car A, i.e., A1_b1 and A1_b2, and Car B, i.e., B1_b1 and B1_b2. The data from Car A is labeled as damaged (D), whereas the data recorded in Car B is labeled as healthy (H). For training and testing, the data was split randomly into datasets of sizes 80,000 and 20,000 samples, respectively. The results are summarized in the form of confusion matrices in Figure 12. While the confusion matrix on the left summarizes the classifications in absolute numbers, the confusion matrix on the right gives the according percentages. It can be observed that the bearing faults can be very well detected with a TNR of 99.69%, while the probability of correctly predicting the healthy bearings is almost as high with 99.50%. Thus, the number of false alarms is negligibly small.

**Figure 13:**
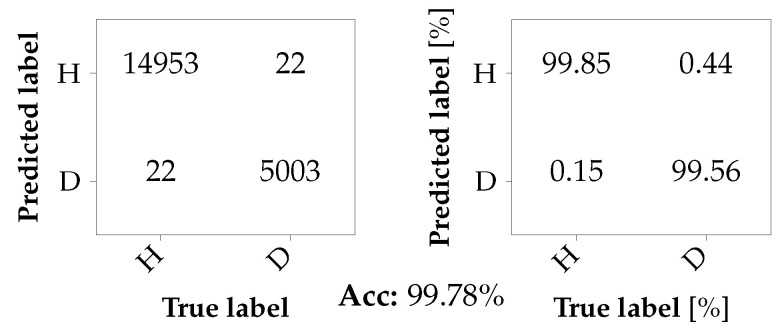
Confusion matrix for a binary classification problem at the gearbox with seen damages.

A similar experiment is conducted for the gearbox. Since only a single instance of bearing damage, i.e., A2_b3, is available for the gearbox, the data obtained from the healthy gearboxes at the three remaining axles, i.e., B1, B2, and A1, are labeled as healthy (H). The sizes of the test and training sets are identical to those in the previous experiment. The resulting confusion matrices are shown in Figure 13. Once again, an almost perfect classification result is achieved, with an accuracy of 99.78%. However, it has to be noted that it cannot be ruled out that the damaged bearing at the motor, i.e., A2_b2, also affects the microphone signal at the gearbox on the same axle and, thus, the classification result. Yet the fact that the classifier is able to correctly classify the data obtained from the healthy gearbox of the first axle of Car *A* as healthy speaks against this concern.

#### 3.4.2. Classification with Unseen Damages

**Figure 14:**
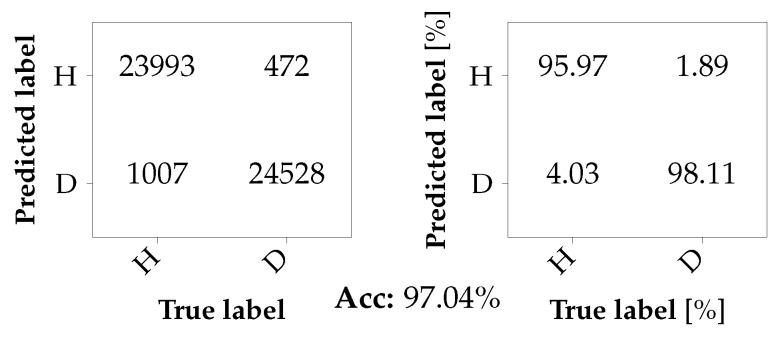
Confusion matrix for a binary classification problem with unseen bearing damage A2_b2 in the test set.

**Figure 15:**
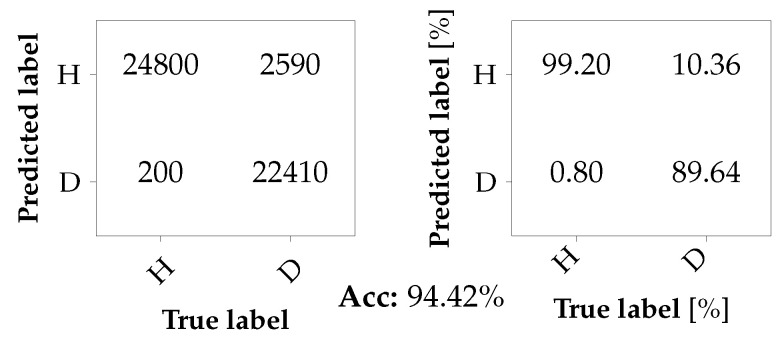
Confusion matrix for a binary classification problem with unseen bearing damage A1_b1 in the test set.

For the classification with unseen damages, the following scenarios are considered: the classifier is trained with data from one of the two damaged bearings at the motor, either A1_b1 or A2_b2, and data from the corresponding axle of Car B, i.e., B1_b1 or B2_b2. The classifier is then tested with data from the remaining damaged bearing and its corresponding healthy reference. Thus, if the classifier is trained with A1_b1 and B1_b1, it is tested with A2_b2 and B2_b2, and vice versa. Again, the training set and the test set consist of 50,000 samples each. Although the scenario in which A2_b2 and B2_b2 form the test set was already briefly discussed in Section 3.3 (cf. Table 6), the complete confusion matrix is now shown in Figure 14. The results for the second scenario where A1_b1 and B1_b1 are used for testing instead of for training are shown in Figure 15. Here, the overall accuracy slightly drops to 94.42%. Noticeably, the TNR has dropped below 90%, which leads to an increased number of missed detections. A plausible explanation for this behavior is the following: while bearing A2_b2 represents a bearing fault in a more advanced stage, bearing A1_b1 represents a fault in a rather early stage. Therefore, it is plausible that when the classifier has only been trained on advanced faults, it has difficulty detecting less severe faults, leading to an increased number of undetected faults.

The experiments with unseen data support the claim that the features are genuinely fault-related rather than axle-related, as the bearing faults at the motor were reliably detected even when positioned on different axles.

In summary, bearing faults can be effectively detected using acoustic data and a feature-based classification approach. However, it is important to note that the available datasets contained only a limited number of bearing faults, which requires careful classifier design to minimize the risk of overfitting.

## 4. Conclusions

In this paper, it is demonstrated that classifying bearing faults in railway vehicles using airborne sound data is feasible even in challenging real-world scenarios. In an experimental study, various features from the time domain and the frequency domain were extracted from sound signals recorded not on a test bench but on a railway vehicle during regular operation and then compared with respect to their performance with various classifiers. This study showed that the Mel Frequency Cepstral Coefficients (MFCCs) clearly outperform all of the other features. In order to optimize the classification performance, the MFCCs were used to train various classifiers. The experiments showed that with our proposed MLP, near-perfect classification results were achieved for scenarios with seen damages, and for unseen data, accuracies exceeded 94%. In conclusion, airborne sound data is well-suited for detecting and classifying bearing faults in the considered scenario. Consequently, microphones can be a valuable addition to acceleration sensors and warrant further investigation. In future work, these promising results should be validated in other scenarios, such as detecting rotor imbalance, and in more complex tasks, like multi-class classification involving various bearing damages, which would require more recorded data. Additionally, exploring a bi-modal approach that combines structure-borne and airborne sound for classification could potentially yield even better results. 

## Figures and Tables

**Table 1:** Description of the damaged bearings at the first two axles of Car A, i.e., A1 and A2 in the field measurements. OR: outer ring fault, IR: inner ring fault, DE: drive-end, NDE: non-drive end, M: motor, G: gearbox. Reprinted with permission from [17].

Bearing	Bearing Type	Damage	Location	Axle	Description
A1_b1	Deep groove ball bearing	IR	DE (M)	A1	Pitting damage
A2_b2	Deep groove ball bearing	OR	DE (M)	A2	Fatigue damage
A2_b3	Cylindrical roller bearing	OR	NDE (G)	A2	Fatigue damage

**Table 2:** Overview of the considered time-domain features in the *TD* feature set.

Feature	Formula
Average $\bar{x}$	$\frac{1}{K-1}\sum_{k=0}^{K-1}x\left[k\right]$
Variance $\sigma_{x}^{2}$	$\frac{1}{K-1}\sum_{k=0}^{K-1}{\left(x\left[k\right]-\bar{x}\right)}^{2}$
Root Mean Square (RMS) $x_{\mathrm{RMS}}$	$\sqrt{\frac{1}{K-1}\sum_{k}^{K}x{\left[k\right]}^{2}}$
Kurtosis	$\frac{\frac{1}{K-1}\sum_{k=0}^{K-1}{\left(x\left[k\right]-\bar{x}\right)}^{4}}{{\left(\sigma_{x}^{2}\right)}^{2}}$
Skewness	$\frac{\frac{1}{K-1}\sum_{k=0}^{K-1}{\left(x\left[k\right]-\bar{x}\right)}^{3}}{{\left(\sqrt{\sigma_{x}^{2}}\right)}^{3}}$
Amplitude range	max(x[k])−min(x[k])
Crest factor	$\frac{\max(|x[k]|)}{x_{\mathrm{RMS}}}$
Clearance factor	$\frac{\max(|x[k]|)}{\sqrt{\frac{1}{K}\sum_{k=0}^{K-1}{\left|x\left[k\right]\right|}^{2}}}$
Impulse factor	$\frac{\max(|x[k]|)}{\frac{1}{K}\sum_{k=0}^{K-1}\left|x\left[k\right]\right|}$
Shape factor	$\frac{x_{\mathrm{RMS}}}{\frac{1}{K}\sum_{k=0}^{K-1}\left|x\left[k\right]\right|}$

**Table 3:** Overview of the considered frequency-domain features in the *FD* feature set. $\mu_{1}=0$ and $\mu_{2}=K-1$ denote the lower and upper frequency bin indices, respectively, and $f_{\mu }$ denotes the frequency corresponding to frequency bin μ. κ is set to 0.95.

Feature	Formula
Spectral centroid (SC)	$\frac{\sum_{\mu =\mu_{1}}^{\mu_{2}}f_{\mu }\left|X\left[\mu \right]\right|}{\sum_{\mu =\mu_{1}}^{\mu_{2}}\left|X\left[\mu \right]\right|}$
Spectral spread (SSpr)	$\sqrt{\frac{\sum_{\mu =\mu_{1}}^{\mu_{2}}{\left(f_{\mu }-\mathrm{SC}\right)}^{2}\left|X\left[\mu \right]\right|}{\sum_{\mu =\mu_{1}}^{\mu_{2}}\left|X\left[\mu \right]\right|}}$
Spectral kurtosis	$\frac{\sum_{\mu =\mu_{1}}^{\mu_{2}}{\left(f_{\mu }-\mathrm{SC}\right)}^{4}\left|X\left[\mu \right]\right|}{{\left(\mathrm{SSpr}\right)}^{4}\sum_{\mu =\mu_{1}}^{\mu_{2}}\left|X\left[\mu \right]\right|}$
Spectral entropy	$\frac{-\sum_{\mu =\mu_{1}}^{\mu_{2}}\left|X\left[\mu \right]|\log(|X\left[\mu \right]|\right)}{\log(\mu_{2}-\mu_{1})}$
Spectral crest	$\frac{\max(|X[\mu ]|)}{\frac{1}{\mu_{2}-\mu_{1}+1}\sum_{\mu =\mu_{1}}^{\mu_{2}}\left|X\left[\mu \right]\right|}$
Spectral roll-off point	$\sum_{\mu =\mu_{1}}^{i}\left|X\left[\mu \right]\right|=\kappa \sum_{\mu =\mu_{1}}^{\mu_{2}}\left|X\left[\mu \right]\right|$

**Table 4:** Classification results for different feature sets. An SVM with RBF kernel was trained with data from B1_b1 (H) and A1_b1 (D) and tested with data from B2_b2 (H) and A2_b2 (D). TPR denotes the accuracy for the healthy class and TNR denotes the accuracy for the damaged class. ACC refers to the overall accuracy.

Feature Set	TPR [%]	TNR [%]	ACC [%]
*TD*	60.38	61.03	**60.71**
*FD*	37.05	72.51	**54.78**
*ENV*	67.63	56.75	**62.19**
*TD* + *FD* + *ENV*	75.48	84.84	**80.16**
*MFCC*	93.57	94.52	**94.05**

**Table 5:** Feature ranking results.

Rank	Method			
	Relief	mRMR	DT	SFS
1	$c_{6}$	$c_{1}$	$c_{6}$	$c_{6}$
2	$c_{7}$	$c_{7}$	$c_{7}$	$c_{7}$
3	$c_{1}$	$c_{6}$	$AMP_{BPFI}$	$c_{1}$
4	$c_{4}$	$c_{13}$	$c_{2}$	$c_{8}$
5	$c_{13}$	$c_{2}$	$AMP_{BPFO}$	$c_{4}$
6	$c_{2}$	$c_{8}$	Spectral roll-off point	$c_{3}$
7	$c_{8}$	$AMP_{RE}$	$c_{8}$	$c_{5}$
8	$c_{3}$	SSpr	Spectral crest	$c_{2}$
9	$c_{5}$	$c_{5}$	$AMP_{CA}$	$c_{13}$
10	$c_{9}$	$c_{12}$	$AMP_{RE}$	$c_{11}$
11	$c_{11}$	$c_{3}$	$c_{4}$	$c_{9}$
12	SSpr	$c_{4}$	$c_{12}$	SC
13	$c_{10}$	$AMP_{BPFO}$	$c_{5}$	SSpr
**ACC [%]**	89.24	90.86	91.71	85.14

**Table 6:** Classification results for different classifiers and *MFCC* feature set consisting of 13 MFCCs and K = 2048 for a binary classification problem with the unseen bearing damage A2_b2 in the test set.

Classifier	TPR [%]	TNR [%]	ACC [%]
*k-NN (k = 5)*	93.00	98.84	**95.92**
*k-NN (k = 10)*	93.72	98.90	**96.31**
*SVM (RBF kernel)*	93.57	94.52	**94.05**
*SVM (lin. kernel)*	91.46	97.43	**94.46**
*SVM (poly. kernel)*	78.66	94.58	**86.62**
*Decision Tree*	86.78	82.91	**84.84**
*Random Forest*	92.52	89.80	**91.16**
*AdaBoost*	93.50	94.24	**93.87**
*Naive Bayes*	92.03	76.04	**84.04**
*LDA*	90.87	99.42	**95.15**
*QDA*	94.47	99.07	**96.77**
*MLP (baseline)*	96.06	94.97	**95.02**
***MLP (proposed)***	**95.97**	**98.11**	**97.04**

## Data Availability

The data supporting the findings of this study are not publicly available due to their proprietary and sensitive nature and are owned by Siemens Mobility GmbH. Data may be made available from the corresponding author upon reasonable request and with permission from Siemens Mobility GmbH.
